# Effects of social prescribing pilot project for the elderly in rural area of South Korea during COVID‐19 pandemic

**DOI:** 10.1002/hsr2.320

**Published:** 2021-07-01

**Authors:** Ji Eon Kim, Yu Lim Lee, Min Ah Chung, Hye Jin Yoon, Dong Eun Shin, Jin Hee Choi, Sangheon Lee, Hae Kyung Kim, Eun Woo Nam

**Affiliations:** ^1^ Healthy City Research Center, Institute of Health and Welfare Yonsei University Wonju South Korea; ^2^ Department of Health Administration Yonsei University Graduate School Wonju South Korea; ^3^ Mirae Campus LINC+ Yonsei University Wonju South Korea; ^4^ Heungeop‐myeon Sub‐Health Center Wonju South Korea; ^5^ Heungeop Community Library Wonju South Korea

**Keywords:** COVID‐19, depression, elderly, rural area, social prescribing

## Abstract

**Background:**

Older adults—classified as a high‐risk group—are highly likely to experience increased loneliness due to the implementation of various policies designed to prevent the spread of COVID‐19. Accordingly, this study aims to examine the effects of a pilot social prescribing project for elderly people in rural area of South Korea during the COVID‐19 pandemic.

**Methods:**

Using the PRECEDE‐PROCEED model, the effectiveness of the pilot project was verified through pre‐ and post‐impact and outcome evaluation.

**Results:**

According to the results of the impact evaluation, *loneliness* reduced significantly, while the *social participation attitude* score increased. Although the average score of *self‐efficacy* increased, it was not statistically significant. Moreover, it was found that *self‐esteem* increased significantly. In the outcome evaluation, *depression* reduced considerably.

**Conclusion:**

To conclude, the pilot social prescribing project was effective in reducing depression and loneliness for the elderly in rural areas of Korea. It was also confirmed that there is potential to develop a new health promotion project that can improve the self‐esteem of the elderly, and expand their social activities. Second, the pilot project was carried out in an integrated manner by utilizing resources in communities with good accessibility. Therefore, it is expected to be used as a new “Integrated community care model” to improve the mental health of the elderly in rural areas. Third, during the COVID‐19 pandemic, the elderly tend to experience increasing feelings of depression, isolation, and loneliness due to “social distancing.” Therefore, it is expected that social prescribing programs for the elderly in rural areas would become a new alternative for relieve mental disorder of the seniors.

## 1 INTRODUCTION

Since COVID‐19's beginning in Wuhan, Hubei Province, China, in December 2019, the virus has now spread worldwide. On March 12, 2020, the World Health Organization (WHO) issued its highest warning level, declaring COVID‐19 “a pandemic”.^1^ The first confirmed case of COVID‐19 was reported in the Republic of Korea on January 20, 2020, when a 35‐year‐old Chinese woman tested positive for the virus. As of September 18, the total number of confirmed cases in Korea was 22 783, and the total number of deaths was 377.^2^ This historically unprecedented pandemic has put individuals in a situation where they are disconnected from one another due to social distancing protocols, the inability to gather in public places, and the closure of educational institutions. According to the WHO, such situations have had a considerable negative effect on people, threatening their mental health.^3^ The U.S. Centers for disease control and prevention (CDC) also reported that such situations attributable to COVID‐19 may induce considerable fear, anxiety, and stress in people, which can develop into symptoms of depression and affect their mental health.^4^ And some research reflected both the physical and psychological pressure of the current COVID‐19 pandemic on people working in medical place.^5^ In particular, older adults—classified as a high‐risk group—are highly likely to experience increased loneliness due to the implementation of various policies designed to prevent the spread of COVID‐19.^6^, ^7^ Accordingly, the need for COVID‐19 management programs for older adults is rising.

In 2016, the UK's National Health Service (NHS) administered a “social prescribing” program to reduce the burden on general practitioners (GPs), and address depression and loneliness among UK citizens. Social prescribing refers to providing a service that goes beyond medical treatment for a disease, linking patients with nonmedical community services.^8^ A new concept based on the belief in social determinants of health, it is thought that health is greatly influenced by socioeconomic factors.^9^ Nonmedical services provided in a community are diverse, including arts, physical activities, learning, volunteering, fellowship meetings, self‐help groups, social security benefits, and educational opportunities.^10^ Professor Andy Haines of the United Kingdom predicted that the physical and mental health of vulnerable groups of people, such as the elderly, would be gravely affected by such situations as the COVID‐19 pandemic, in which social anxiety increases, and people are further isolated. Haines argued that social prescribing, while utilizing community resources would be an effective way to address the problem.^11^ A study reported that a social prescribing program using community resources could be an option when the fatigue of healthcare personnel is rising from the prolonged COVID‐19 pandemic.^12^ In Ontario, Canada, social prescribing guidelines and programs to cope with COVID‐19 have been newly formulated,^13^ whereas the UK is implementing social prescribing programs centered around community NGOs.^14^ In Korea, it has been suggested that it is necessary to develop a “Social prescribing program” that includes strategies such as keeping social distance, tele counselling in a pandemic era and online distance learning for health education. Taiwan is also discussing social prescribing as a way to cope with the pandemic.^15^ It is thus clear that social prescribing programs for people suffering from the impact of COVID‐19 are being developed worldwide.

Accordingly, this study was conducted to identify the effectiveness of the social prescribing model in the COVID‐19 pandemic era by investigating its impact on depression, loneliness, and attitudes toward social participation, self‐efficacy, and self‐esteem in elderly people in South Korea.

## 2 METHODS

### 2.1 Study design

**FIGURE 1:**
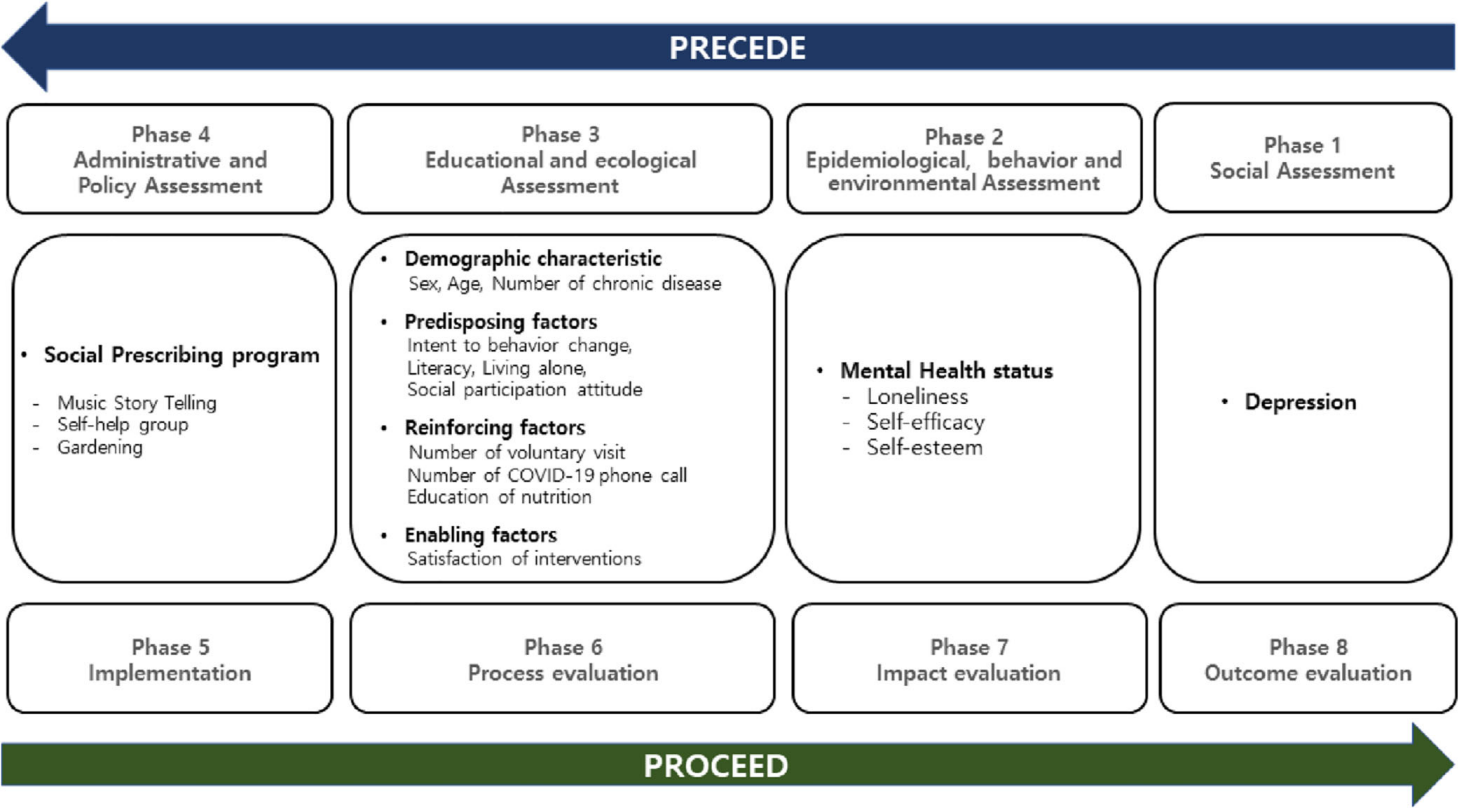
Research design

It was hypothesized that the interventions administered in the pilot social prescribing project would improve subjects' health status by modifying environmental and ecological factors. The effectiveness of the pilot project was tested by utilizing the PRECEDE‐PROCEED model, and conducting pre‐and post‐impact evaluations (Figure 1).^16^

### 2.2 Interventions

The interventions were administered once a week for approximately 10 weeks from May 1 to July 10, 2020. The specific contents of the interventions are shown in Table 1. The interventions consisted of three parts: music storytelling, a self‐help group, and gardening. Two coordinators participated in all intervention programs, and interventions for the program were applied to the subjects in a parallel method.

**TABLE 1:** Intervention activities in social prescribing program

Intervention	Activities	Location	Time
Music storytelling	RFL (rhythm for life)	Community library	One per a week (90 minutes)
	GIM (guide image music)		
	MIC (movement improvisation circle)		
Self‐help group (School of Hunjang)^a^	Healthy 100‐year lecture: fruit	Community library	One per a week (60 minutes)
	Making natural soup for COVID‐19 prevention		
	Making masks for COVID‐19 prevention		
	Making natural diffuser		
	Song writing for community		
Gardening	Education of gardening in spring and summer	Community garden	One per a week (30 minutes)
	Nursery plant (cherry tomato, sweet potato, corn, pepper)		
	Monitoring and managing community garden		
	Harvesting		
COVID‐19 prevention	Voluntary visit (per a week)	‐	Three per a week
	COVID‐19 phone call (per a week)		Four per a week
	Education of healthy nutrition in COVID‐19 pandemic		One per a week

^a^ *Hunjang*: The teacher or headmaster of the seodang was called the *hunjang*.

The range of social prescribing services provided by the UK's NHS National Social Prescribing Network includes interventions of music, sports, arts, clubs, green activities, volunteering, learning, education, and so on (Karen et al., 2017).^17^, ^18^ The Music Storytelling program is a social prescribing that improves people's quality of life and alleviates mental health through artistic intervention in health (Philip et al., 2019).^19^, ^20^ Green activity in Social Prescribing program is such as gardening and horticulture, and through contact with nature, people become healthier physically and mentally (Karen et al, 2017).^18^ Self‐help groups and volunteering, learning, and education programs have clear health benefits, and support for each other can increase social connectivity (Lee & Brudney, 2008; NHS England & NHS Improvement, 2016).^21^, ^22^, ^23^

#### 2.2.1 Music storytelling

Music storytelling refers to discovering stories that individuals have within through music therapy techniques to help them live better lives.^24^ The contents of the music storytelling program are shown in Table 2. Including activities to strengthen seven domains (ie, cognition, sensation, language, physical functioning, ego, mind and body, and sociability), the program was led by two experts with the assistance of 17 university student volunteers. The volunteers were recruited from three universities located in the study area, and received 1‐week training prior to their participation. The music storytelling program was offered in the Heungeop community Library for 90 minutes every Wednesday, from May 1 to July 10, 2020.

**TABLE 2:** Music story telling program

Time	Theme	RFL (rhythm for life)	GIM (guide image music)	MIC (movement improvisation circle)
1 to 3 Week	“I”	Rhythm of self‐introduction	“I”	Healthy clapping
		Rhythm of self‐presentation	Making a story book (praise for completion)	Body tapping, massage
		Rhythm of my hometown	Expressing my hometown	Healthy breathing
4 to 6 Week	“You”	Rhythm of improvisation	“You”	Healthy clapping
		Imitative and open Rhythm	Making a story book (drawing hand of my friend)	Healthy gymnastics (Hand in hand)
		Expressing Rhythm with friends	Pleasure moments with friends	Healthy gymnastics (I like you)
7 to 9 week	“We”	Rhythm of our hometown	“Our town”	Moving as a group
		Song writing with “A‐ri‐rang” melody	Making a story book (10 rainbow healthy life rules)	Massaging with each other
		Making a song of our town using “A‐ri‐rang” melody	Making a knot with each other	Dance a Waltz with each other
10 week	Active	Group activity presentation (Ganggangsuwolrae)	Making a story book (with Korean Flag)	Group dance

#### 2.2.2 Self‐help group

The goals of self‐help groups are to provide older adults with mutual aid rather than a one‐directional service benefit, and establish a social support system. Self‐help groups have a positive impact on the self‐esteem of older adults and the formation of social networks.^25^ In this study, a self‐help group was organized by utilizing the Heungup Township Community Library, one of the resources available in the study area. The group met for 60 minutes every Friday, and participants were encouraged to attend the program voluntarily. Program activities included making natural soaps, facemasks for the prevention of COVID‐19, and creating natural diffusers. Additionally, songs for healing were written for the community suffering due to COVID‐19, and education on healthy fruits and diets was provided under the theme of “stay healthy until the age of 100.” To improve understanding and keeping in mind the elderly participants' literacy levels, figures and photographs were utilized in the educational and nutritional material distributed.

#### 2.2.3 Gardening

The American Horticultural Therapy Association (AHTA) defined horticultural therapy as the process of enhancing individuals' social, psychological, and physical adaptabilities through plants and gardening activities to promote physical, mental, and spiritual healing.^26^ In this study, a community garden was created in collaboration with the Korea National Agricultural Extension Center, a community resource. A feeling of achievement was fostered among participants through productive activities such as growing sweet potatoes, peppers, cherry tomatoes, and corn. The program was conducted every week on Fridays, and participants were instructed to manage the garden in partnership with university student volunteers.

### 2.3 Study subjects

The program participants were 16 adults aged 65 or older, recommended by the Administrative and Welfare Office in a rural area in Gangwon Province. They consented to participate in the study's interventions over 10 weeks. Of the 16 participants, 10 attended all 10 program sessions, and these participants were selected as the study subjects. Arrangements were made for the subjects to visit a public health doctor at the community public health center for one‐on‐one, in‐person examinations pre‐ and post‐intervention. The doctor's medical statement contained information on clinical health status (including underlying disease), a neuropsychiatric evaluation regarding mood disorders and dementia status, and a physician's opinion on future treatment.

### 2.4 Data collection and the description of variables

The survey used in this study was comprised of items addressing socio‐demographics, depression, loneliness, social participation, self‐efficacy, and self‐esteem. The reliability and validity of the instruments were analyzed by a PhD in public health and a statistician. The survey was administered twice, before and after the interventions. To administer the survey, 13 survey takers were recruited and trained by the PhD student and statistician on the survey items and the administration method over 2 days, for approximately, 1 hour per day. The trained survey takers met in person with the subjects to whom they were allocated, and administered the survey in a one‐on‐one interview setting. The survey took an average of 15 minutes to complete.

#### 2.4.1 Depression

As an instrument to measure the change in depression pre‐ and post‐intervention, the geriatric depression scale (GDS) was chosen,^27^ and the Geriatric Depression Scale‐Korean Version (GDS‐K) was used,^28^ keeping in mind the Korean‐speaking elderly subjects of the study. The GDS‐K consists of 30 items, and a score under 13 points is classified as normal, whereas a score of 14 to 18 indicates mild depression, a score of 19 to 21 indicates moderate depression, and a score over 22 signifies severe depression. The value of Cronbach's *α*, an index for reliability, was .84 in the GDS‐K validation study and .82 in this study, suggesting that the GDS‐K is a reliable instrument.

#### 2.4.2 Loneliness

Loneliness was assessed using a scale revised from the UCLA Loneliness Scale translated into Korean.^29^ Ten items that fit in with Korea's situation, five positive and five negative, were used in this study. The instrument used in the study had a Cronbach's *α* value of .70.

#### 2.4.3 Social participation attitude

To examine the level of older adults' social participation in a variety of interpersonal relationships, the social participation scale for the elderly living alone was used.^30^ The instrument consists of eight items evaluated on a five‐point scale. The Cronbach's *α* of the instrument was .90 in a previous study, and .85 in the present study.

#### 2.4.4 Self‐efficacy

Self‐efficacy refers to a person's belief in his or her competence to perform successfully in producing a certain outcome.^31^ To assess self‐efficacy, the Korean Adaptation of the General Self‐Efficacy Scale was used; it was based on the general self‐efficacy scale (GSE) developed by Schwarzer,^32^ and tested for internal reliability.^33^

#### 2.4.5 Self‐esteem

Self‐esteem is the evaluative element in self‐perception and refers to a person's acceptance of self as a positive and worthwhile being.^34^ In this study, self‐esteem was assessed with a Korean translation of the Rosenberg Self‐Esteem Scale.^35^, ^54^ The instrument consists of 10 items—five regarding positive self‐esteem and five regarding negative self‐esteem. All items were evaluated on a five‐point scale. Scores range from 10 to 50 and the higher the score, the higher one's self‐esteem. Cronbach's *α* for the entire instrument was .84.

### 2.5 Statistical analysis

The data were analyzed using SPSS Statistics (25.0 version). A Wilcoxon signed‐rank test was conducted for impact and outcome evaluations of the social prescribing programs. Specifically, the statistical analysis proceeded as follows. First, means and standard deviations were computed, and a frequency analysis was performed to examine subjects' sociodemographic, epidemiological, behavioral, and environmental factors, and assess the predisposing, reinforcing, and enabling factors. Second, the Wilcoxon signed‐rank test was conducted to examine changes before and after implementation of the social prescribing programs with respect to depression, loneliness, social participation attitude, self‐efficacy, and self‐esteem.

### 2.6 Ethical approval

This research protocal has been reviewed and approved by the Institutional Review Board (IRB) of Yonsei University Mirae Campus (number: 1041849‐202 010‐SB‐146‐04).

## 3 RESULTS

### 3.1 PRECEDE assessment before implementation of social prescribing programs

Results from the PRECEDE assessment phases are shown in Table 3. First, regarding the social assessment phase, all 10 subjects were female (100%). The mean age was 82.0 years, with six subjects (60.0%) responding that they were over 80, and four (40.0%) responding that they were under 80. Regarding the number of chronic illnesses, three was the most common answer (70.0%), followed by one (20.0%) and two (10.0%). Regarding the score for depression measured using the GDS‐K, six subjects (60.0%) scored 13 or lower (classified as normal); this category was the largest. The second largest category was mild depression, with two subjects (20.0%) whose scores were between 14 and 18, followed by one subject (10.0%) each falling in the categories of moderate depression (19‐21) and severe depression (22 and up).

**TABLE 3:** PRECEDE assessment before social prescribing program (N = 10)

Category			N	%
Social assessment	Demographic characteristics			
	Sex	Female	10	100.0
	Age	<80	4	40.0
		≥80	6	60.0
		Average	82.0 (SD 5.9)	
	Number of chronic disease	1	2	20.0
		2	1	10.0
		≥ 3	7	70.0
	Depression (GDS‐K)			
	GDS‐K score	≤13	6	60.0
		14 ≤ 18	2	20.0
		19 ≤ 21	1	10.0
		≥22	1	10.0
Epidemiological, behavior, and environmental assessment	Mental health status			
	Loneliness	≤24	4	40.0
		25 ≤ 29	5	50.0
		≥30	1	10.0
	Self‐efficacy	≤22	5	50.0
		23 ≤ 26	3	30.0
		≥28	2	20.0
	Self‐esteem	≤31	3	30.0
		32 ≤ 36	6	60.0
		≥37	1	10.0
	Diagnosis from doctor			
	DSM‐5 (major depressive disorder)	Yes	1	10.0
		No	9	90.0
	K‐MMSE (cognitive function decline)	Yes	2	20.0
		No	8	80.0
Predisposing factors	Social participation attitude	≤ 31	3	30.0
		32 ≤ 36	6	60.0
		≥ 37	1	10.0
	Literacy	Yes	3	30.0
		No	7	70.0
	Living alone	Yes	9	90.0
		No	1	10.0
Reinforcing factors	Voluntary visit (per a week)	Yes	0	0.0
		No	10	100.0
	COVID‐19 phone call (per a week)	Yes	0	0.0
		No	10	100.0
	Education of healthy nutrition	Yes	0	0.0
		No	10	100.0
Enabling factors	Scale of happiness (total score: 100)	Average	54.40 (SD 6.9)	

In the epidemiological, behavioral, and environmental assessment phases, the scores for loneliness, self‐efficacy, and self‐esteem, as well as the *Diagnostic and Statistical Manual of Mental Disorders* (DSM‐5), and the *Korea Mini‐Mental State Examination* (K‐MMSE) test results, were analyzed. Regarding loneliness, the majority of subjects (n = 5, 50.0%) had a score between 25 and 29. The second most common score range was under 24 (n = 4, 40.0%), followed by a score over 30 (n = 1, 10.0%). In regard to self‐efficacy, the majority of subjects (n = 5, 50.0%) had a score under 22, followed by scores ranging from 23 to 26 (n = 3, 30.0%), and over 28 (n = 2, 20.0%). With regard to self‐esteem, the most common score range was 32 to 36 (n = 6, 60.0%), followed by a score under 31 (n = 3, 30.0%) and over 37 (n = 1, 10.0%). Regarding the DSM‐5 (major depressive disorder) test results, nine subjects (90.0%) were classified as “normal,” and one subject was classified as “requiring consultation with a psychiatrist.” Lastly, regarding the K‐MMSE (dementia status) test results, eight subjects (80.0%) were classified as normal, and two subjects (20.0%) as requiring consultation with a psychiatrist for cognitive competence.

For predisposing factors, social participation attitude and literacy were examined, as well as whether a subject lived alone. Regarding the score for social participation attitude, the largest group of subjects (n = 6, 60.0%) scored between 23 and 36. The second common score range was under 31 (n = 3, 30.0%) and over 37 (n = 1, 10.0%). In terms of literacy, seven subjects (70.0%) responded that they were illiterate; there were thus more illiterate subjects than literate subjects (n = 3, 30.0%). Of the 10 subjects, nine (90.0%) lived alone, and one subject (10.0%) lived with family members. For reinforcing factors, the following was chosen: the (weekly) number of visits by volunteers, the (weekly) number of phone calls regarding COVID‐19 prevention, and the number of times the subject received nutritional education. It was found that none of the subjects had these experiences. As an enabling factor, satisfaction with the social prescribing programs was examined using a scale for happiness before and after intervention. The mean happiness score was 54.40 (SD 6.9).

### 3.2 Progress evaluation after social prescribing programs

Results from the progress evaluation phase pre‐ and post‐implementation of the social prescribing pilot project are shown in Table 4. In the study, three social prescribing programs were executed as interventions, namely music storytelling, a self‐help group, and gardening. For reinforcing factors, the (weekly) number of visits by volunteers, the (weekly) number of phone calls regarding COVID‐19 prevention, and the number of times the subjects received nutritional education during the duration of the interventions were selected. None of the subjects had experienced any of the reinforcing factors prior to the intervention. After the interventions, the (weekly) number of visits by volunteers was three, the (weekly) number of phone calls regarding COVID‐19 prevention was four, and the number of sessions for nutritional education was six. As an enabling factor, satisfaction with the social prescribing programs was evaluated by measuring the level of happiness pre‐ and post‐intervention; for the predisposing factor, the attitude toward social participation was measured. The mean happiness score was 54.40 (SD 6.9) before intervention, and 83.20 (SD 6.4) after. Mean scores for the attitude toward social participation were 29.90 (SD 5.7) and 35.10 (SD 3.3) before and after intervention, respectively.

**TABLE 4:** Progress evaluation after social prescribing program (N = 10)

Category			Before	After
			M ± SD	
Reinforcing factors	Number of voluntary visit (per a week)		0	3
	Number of COVID‐19 phone call (per a week)		0	4
	Education of healthy nutrition		0	6
Enabling factors	Satisfaction of social prescribing program	Scale of happiness (total score: 100)	54.40 (SD 6.9)	83.20 (SD 6.4)
Predisposing factors	Social participation attitude change		29.90 (SD 5.7)	35.10 (SD 3.3)
Progress evaluation	Mental health	Loneliness	25.60 (SD 2.5)	22.20 (SD 5.0)
		Self‐efficacy	23.40 (SD 4.8)	27.00 (SD 6.3)
		Self‐esteem	31.80 (SD 4.2)	38.40 (SD 6.3)
		Depression (GDS‐K)	13.60 (SD 5.0)	10.00 (SD 3.7)

In the progress evaluation phase, subjects' characteristics were evaluated. According to the findings, the mean loneliness score was 25.60 (SD 2.5) before intervention, and 22.20 (SD 5.0) after. Regarding self‐efficacy, the mean pre‐intervention score was 23.40 (SD 4.8), and the mean post‐intervention score was 27.00 (SD 6.3). Regarding self‐esteem, the mean pre‐intervention score was 31.80 (SD 4.2), and the mean post‐intervention score was 38.40 (SD 6.3). Lastly, the mean pre‐intervention depression score, measured with the GDS‐K, was 13.60 (SD 5.0), and the mean post‐intervention score was 10.00 (SD 3.7).

### 3.3 Impact and outcome evaluation after the social prescribing project

**FIGURE 2:**
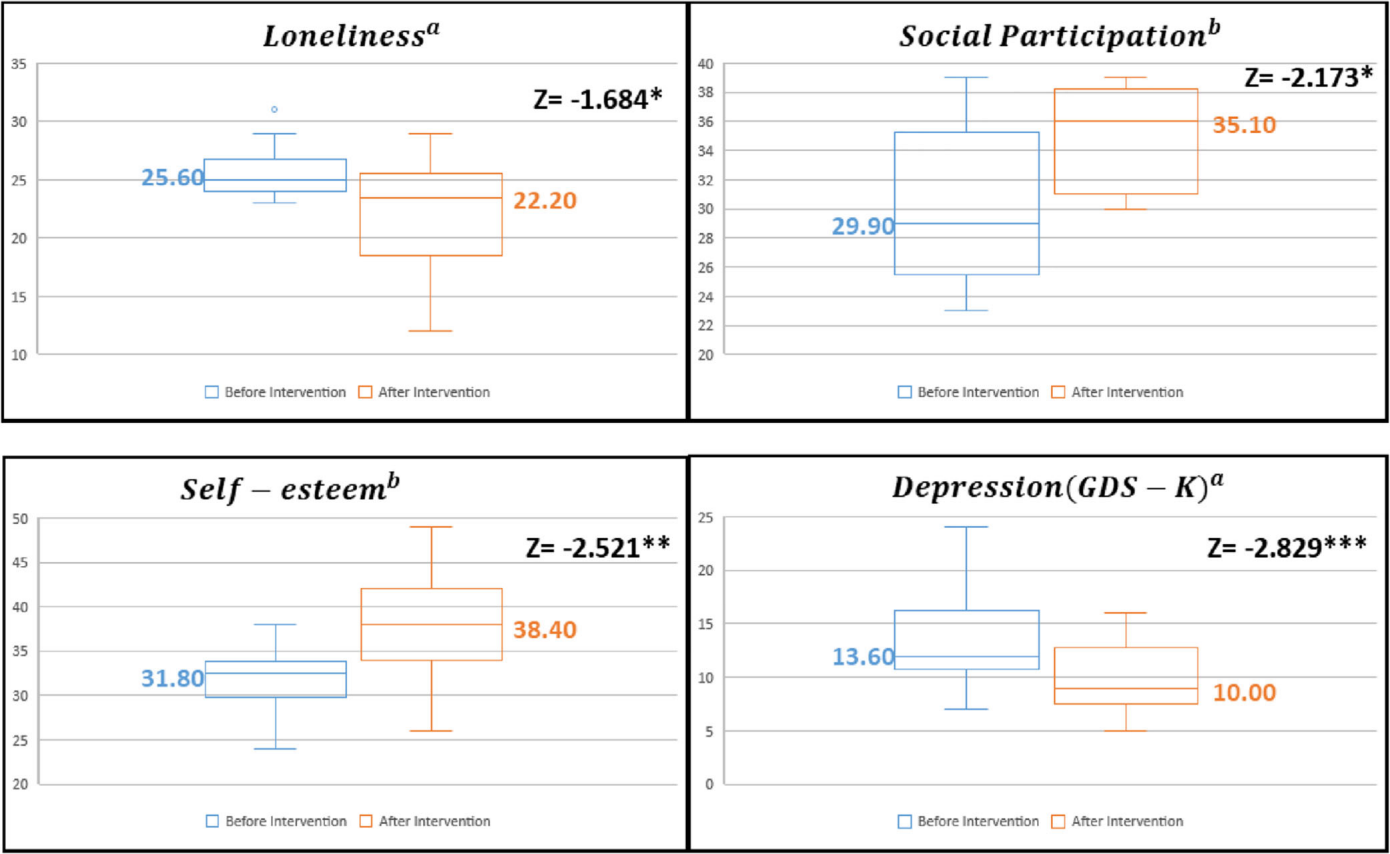
Impact and outcome evaluation boxplot

The Wilcoxon signed‐rank test was performed to evaluate the pre‐ and post‐impact and outcome of the pilot social prescribing project and the results are presented in Table 5. To evaluate the impact of the pilot project, pre/post changes in loneliness, social participation attitude, self‐efficacy, and self‐esteem were analyzed. First, loneliness tended to decrease statistically (*Z* = −1.684, *P* < .1). Furthermore, the attitude toward social participation statistically and significantly increased (Z = −2.173, *P* < .05) after intervention. The mean self‐efficacy score improved post‐intervention, although the increase was not statistically significant. Self‐esteem was statistically and significantly elevated (Z = −2.521, *P* < .05) after intervention. For the outcome evaluation of the pilot project, the change in depression was examined, and it was found that depression had significantly decreased (Z = −2.829, *P* < .01; Figure 2).

**TABLE 5:** Impact and outcome evaluation after social prescribing program (N = 10)

Category			N	Mean Rank	Sum of Ranks	*z*
Impact evaluation	Loneliness^a^	(−) Rank	7	6.29	44.00	−1.684*
		(+) Rank	3	3.67	11.00	
		Ties	0			
	Social participation^b^	(−) Rank	1	2.50	2.50	−2.173**
		(+) Rank	7	4.79	33.50	
		Ties	2			
	Self‐efficacy^b^	(−) Rank	3	4.67	14.00	−1.011
		(+) Rank	6	6	31.00	
		Ties	1			
	Self‐esteem^b^	(−) Rank	0	0	0	−2.521**
		(+) Rank	8	4.5	36.00	
		Ties	2			
Outcome evaluation	Depression (GDS‐K)^a^	(−) Rank	10	5.50	55.00	−2.829***
		(+) Rank	0	0	0	
		Ties	0			

^a^ Reference: (+) Rank.

^b^ Reference: (−) Rank.

* *P* < .1.

** *P* < .05.

*** *P* < .01.

## 4 DISCUSSION

For the subjects who participated in all three social prescribing intervention programs (music storytelling, the self‐help group, and gardening), scores for both depression and loneliness decreased statistically and significantly, whereas social participation attitude and self‐esteem statistically and significantly increased. Previous studies have found that social prescribing programs had the effect of lowering depression,^36^, ^37^ and, along with related activities, were effective in easing loneliness and the feeling of isolation in older adults.^38^, ^39^, ^40^ In addition, according to Munford,^10^ who studied the effect of a social prescribing program for adults aged 65 or older with a chronic illness in Salford, northern England, and the use of a community‐based social prescribing program enhanced their overall quality of life, positive moods, interpersonal relationships, and self‐esteem. It also lowered helplessness and fatigue indices. In particular, social prescribing programs that are developed utilizing community assets, and designed to fit the unique features of the community and individual situations, have better potential for success.^41^ In the present study, the social prescribing program was executed for older adults in a rural area using community assets, such as the Community Library, and positive findings were obtained (similar to those of previous studies). Additionally, each subject's health status was examined by a public health doctor before and after intervention, and subjects participated in the program based on the doctor's recommendation. Accordingly, the study was structured similarly to the UK's social prescribing model.^17^ In social prescribing programs, patients are diagnosed by a doctor, and customized social prescriptions are supported through a social prescription coordinator (Link Worker). Primary care clinician makes a social prescription—a referral—after fulsome discussions and connected to social and community supports. These social prescribing programs can reduce the burdensome work of doctors through connection to nonpharmaceutical treatment in the community rather than drug treatment, and can be effective in solving chronic diseases and mental health problems. In addition, it can be a way to reduce the burden of medical expenses due to excessive medical use.^42^

On the other hand, it has been stated that for a social prescribing intervention program to be successful, the program should generally involve subjects' productive participation and transformation, a community‐based approach, and should be implemented in an integrative manner.^43^ The social prescribing intervention programs in this study had all of these features. Regardless of how beneficial an activity might be, if depressed lonely older adults perceive it as something for “lonely older people,” they immediately regard it as an undesirable or unhelpful service, and their participation rate drops precipitously.^44^ The participation rate was high in the present study because opportunities for voluntary participation were made available to study subjects through the self‐help group and programs that were familiar to them, owing to the active use of community resources. Further, as subjects were engaged in productive outdoor activities via the gardening program, interpersonal relationships improved, while self‐esteem and feelings of achievement increased. One of the primary effects of horticultural therapy and gardening is increased self‐esteem. Goals of music storytelling include the reinforcement of cognitive competence and sensations, linguistic competence improvement, a calm mind and body, increased sociability, self‐discovery, and enhanced physical functioning. In the present study, music and stories were combined, and psychological counselling techniques were utilized in music storytelling. A previous study found that a music storytelling program for older adults in rural areas had the effects of “joy from participating,” “vitality from intergenerational exchanges,” and “expanded interpersonal relationships”.^37^ Another study reported that social prescribing and the use of music had the positive impacts of reducing stress and increasing social participation.^19^ Based on the current study findings, it seems that a social prescribing program is effective in lessening depression and loneliness in older adults in rural areas; additionally, self‐esteem is positively influenced and social participation can be expected to increase. Accordingly, the findings show that it would be feasible to develop a social prescribing program for a new rural‐type health promotion project in South Korea.

Social prescribing can be an option to manage the mental health of rural community residents during the COVID‐19 pandemic.^45^ The WHO recommends that efforts be made to stay physically “active” in order to maintain physical and mental capacities for recovery, even though the COVID‐19 situation is worsening.^46^ In the COVID‐19 pandemic, older adults are experiencing heightened feelings of depression, loneliness, and social isolation from being alone.^47^ The United Kingdom is providing social prescribing interventions to strengthen primary healthcare during the COVID‐19 pandemic by utilizing community health and welfare workers.^48^ In this study too, social prescribing programs were implemented during the pandemic, and such activities as the transmission of accurate information on COVID‐19 via promotional data, social prescribing through telecommunication (via telephone), and the delivery of COVID‐19 protection gear were supported. It was anticipated that a social prescribing program would be more effective in a crisis because of its basis: an asset‐based community development (ABCD) model approach, formulated according to the theory of salutogenesis, integrated with customized nonmedical service.^45^, ^49^ As the COVID‐19 pandemic persists, many complications have been arising, to the extent of coining the term “Corona Blues”.^50^ Policy solutions, however, are lacking. The US CDC stated that a conscious effort to reinforce communities would be needed to cope with stress and loneliness resulting from COVID‐19,^4^ and many studies warn that drinking, smoking, and drug use may further exacerbate depression.^51^, ^52^, ^53^ Accordingly, to solve the mental health problems of older adults in rural areas during the COVID‐19 pandemic, it is necessary to formulate an integrative community care model for social prescribing, appropriate for the current pandemic situation in the use and linking of community assets, while public health centers responsible for primary healthcare and Administrative and Welfare offices also play a central role. On the basis of the study results, it is believed that such social prescribing programs can provide an effective psychological defense for the rural elderly, who may tend to become easily withdrawn as indoor activity increases because of the social distancing policy for COVID‐19 prevention.

There are also several limitations in this study. First, this study only analyzed participants who attended all 10 weeks in the social prescribing program. Because the high average of the subjects led to manage and coordinate the attendance difficulty. Therefore, it is necessary to consider how to manage the dropout rate of research subjects in future social prescription programs. Second, since research related to social prescribing is a process that requires finding an integrated basis for a wide variety of intervention programs, not only quantitative studies but also qualitative studies are important.^43^ Therefore, in future studies, it is necessary to evaluate the effectiveness using a research method that combines not only quantitative studies but also qualitative studies. Third, the social prescribing pilot project conducted in Korea left some weaknesses. This study is small‐scale, does not have a control group, much of the evidence available is qualitative, and relies on self‐reported outcomes. However, there is a growing body of evidence that social prescribing can lead to a range of positive health and wellbeing outcomes. If the social prescribing project is expanded in integrated with local communities and local social enterprises, more sophisticated scientific research will be possible.

## 5 CONCLUSION

First, the social prescribing programs were effective in reducing depression and loneliness in elderly people in rural area. Additionally, it was confirmed that it is feasible to develop such programs for a new health promotion project in which self‐esteem is positively influenced, and greater social participation of older adults can be expected.

Second, the pilot social prescribing project was executed in an integrative manner, utilizing community resources with excellent accessibility. Hence, it is anticipated that the project could be used as a new integrated community care model to promote mental health in elderly people in rural area.

Third, as indoor activity increases because of social distancing and other policies in the COVID‐19 pandemic era, it is expected that social prescribing programs would be a new alternative to equip elderly people in rural areas—who would typically withdraw from social participation and feel increasingly more depressed, isolated, and lonely—with an effective psychological defense.

## CONFLICT OF INTEREST

All authors declare that they have no conflict of interest to declare.

## AUTHOR CONTRIBUTIONS

Conceptualization: Ji Eon Kim, Eun Woo Nam, Dong Eun Shin

Data Curation: Si Chen, Yan Wang

Funding Acquisition: Eun Woo Nam, Jin Hee Choi

Investigation: Ji Eon Kim, Yu Lim Lee, Min Ah Chung, Hye Jin Yoon, Sangheon Lee

Methodology: Ji Eon Kim, Eun Woo Nam

Project Administration: Eun Woo Nam, Hae Kyung Kim

Resources: Ji Eon Kim, Eun Woo Nam, Dong Eun Shin

Supervision: Eun Woo Nam

Writing—Original Draft Preparation: Ji Eon Kim

Writing—Review & Editing: Ji Eon Kim, Eun Woo Nam

  All authors have read and approved the final version of the manuscript.

  Eun Woo Nam had full access to all of the data in this study and takes complete responsibility for the integrity of the data and the accuracy of the data analysis.

## TRANSPARENCY STATEMENT

The lead author affirms that this manuscript is an honest, accurate, and transparent account of the study being reported; that no important aspects of the study have been omitted; and that any discrepancies from the study as planned (and, if relevant, registered) have been explained.

## Data Availability

The authors confirm that the data supporting the findings of this study are available within the article supplementary materials.
